# Lessons from an evaluation of an urban Indigenous food sharing initiative in Southwestern Ontario: “I feel like I’m nourishing my spirit”

**DOI:** 10.17269/s41997-025-01044-2

**Published:** 2025-05-19

**Authors:** Laura Peach, Kaitlyn Patterson, Dave Skene, Sarina Perchak, Kelly Skinner, Hannah Neufeld

**Affiliations:** 1 School of Public Health Sciences, University of Waterloo, Waterloo, ON Canada; 2 Wisahkotewinowak Collective, Kitchener, ON Canada; 3 Department of Public Health Sciences, Queen’s University, Kingston, ON Canada

**Keywords:** Indigenous, Urban, Food support, Health, Wellbeing, Program evaluation, Community-based participatory research, Autochtone, Urbain, Soutien alimentaire, Santé, Bien-être, Évaluation de programme, Recherche participative basée sur la communauté

## Abstract

**Objectives:**

The aim of this study was to examine an urban Indigenous food sharing initiative through an evaluation attending to the Food Share Initiative’s implementation and early outcomes.

**Methods:**

This project used a community-based participatory research methodology to guide an evaluation of process and initial outcomes. Storytelling methods including interviews and a sharing circle, which took place in July and August 2021, were used to create a relational context for the research team and project participants, which honour Indigenous research methodologies.

**Results:**

A total of 14 self-identifying Indigenous people participated in this evaluation. Initiative staff and Food Share recipients identified community relationships as a shared initiative experience that contributed to the wholistic health effects experienced by recipients. All participants recognized capacity limitations of both Food Share recipients and operational staff were important constraints to the initiative’s process and implementation. Participant recommendations to improve the Food Share included enhanced outreach to involve other Indigenous community members as well as infrastructure like long-term funding and a central location to strengthen the initiative’s operational capacity.

**Conclusion:**

As an important community food support, the Food Share’s relational care approach fosters a meaningful and wholistic sense of nourishment for Indigenous community members in the Waterloo-Wellington Region.

## Résumé

**Objectifs:**

Étudier une initiative autochtone de partage des aliments en milieu urbain, la Food Share Initiative, au moyen d’une évaluation de sa mise en oeuvre et de ses premiers résultats.

**Méthode:**

Une méthode de recherche participative basée sur la communauté a guidé l’évaluation du processus et des premiers résultats de l’initiative. Nous avons utilisé des méthodes de mise en récit, dont des entretiens et un cercle de partage, en juillet et en août 2021 afin d’aménager pour l’équipe de recherche et les participants du projet un cadre relationnel qui honore les méthodes de recherche autochtones.

**Résultats:**

Quatorze personnes s’identifiant comme étant autochtones ont participé à l’évaluation. Selon le personnel et les bénéficiaires de Food Share, l’expérience commune des liens communautaires établis dans le cadre de l’initiative a contribué à ses effets holistiques sur la santé des bénéficiaires. Toutes les personnes participantes ont reconnu que les moyens limités des bénéficiaires et du personnel opérationnel de Food Share constituaient une contrainte importante de son processus et de sa mise en oeuvre. Leurs recommandations pour améliorer Food Share étaient de faire rayonner l’initiative auprès des membres d’autres communautés autochtones et d’en améliorer les infrastructures en lui accordant un financement à long terme et un emplacement central pour renforcer sa capacité opérationnelle.

**Conclusion:**

Food Share est un important organisme de soutien alimentaire de proximité, et son approche axée sur les soins relationnels engendre chez les membres des communautés autochtones de la région de Waterloo-Wellington un profond sentiment d’être nourris dans leur globalité.

## Introduction

For millennia, Indigenous Peoples on Turtle Island (otherwise known as North America) have had strong connections with their local food systems, and these relationships have supported wholistic health, including physical, mental, emotional, and spiritual wellbeing (Malli et al., 2023). Organizing food practices according to the Earth’s natural order and alongside cultural values of relationality, responsibility, respect, and reciprocity enables Indigenous Peoples to maintain traditions that support sacred food systems, while strengthening food security (i.e. sufficient access to safe, nutritious, and culturally appropriate food) and promoting individual, community, land, and territorial health (Morrison, 2011). Yet, centuries of settler colonialism in Canada have threatened Indigenous Peoples’ relationships with their food systems by systematically displacing Indigenous Peoples from their lands and waters; damaging Indigenous ecologies through extraction, development, and pollution leading to biodiversity loss and unsafe food supplies; imposing heavy regulations on subsistence activities and forcing reliance on wage labour and market foods; and disrupting land-based intergenerational knowledge transfer within families and communities (Timler et al., 2019). The cumulative effects of colonialism have created socioeconomic inequities that produce disproportionate rates of food insecurity among Indigenous Peoples and increase the risk of adverse health outcomes, including non-communicable diseases such as diabetes (Malli et al., 2023) .

Experiences of food insecurity among Indigenous Peoples intensified in the context of the COVID-19 pandemic. Notably, Indigenous adults were more likely to experience food-related worry than their Canadian counterparts in 2020 (Daly et al., 2023), and in 2022, one third (33.4%) of Indigenous individuals living off-reserve across the ten provinces within Canada experienced household food insecurity (a nearly 3% increase in just one year) (Li et al., 2023). Although limited in number, studies that explore food insecurity levels within urban Indigenous communities during the COVID-19 pandemic report significant increases and crisis levels (Richmond & Dokis, 2023; Shafiee et al., 2023; Watson et al., 2022). Unique barriers to food security for Indigenous Peoples in cities include limited cultural supports (including limited access to Indigenous foods) and higher costs of living (Skinner et al., 2016), and both issues are exacerbated by isolation measures and income disruptions occurring within the pandemic (Watson et al., 2022).

Persistent and rising rates of food insecurity among Indigenous Peoples stem from colonialism and are compounded by other structural and social determinants of health. As such, there is a need for Indigenous-determined, community-initiated, and place-based strategies that make progress towards greater food sovereignty and protect Indigenous foodways as well as community and cultural connections in support of wholistic health and wellbeing (Timler et al., 2019). The concept of Indigenous food sovereignty (IFS) represents a way of knowing and doing through which Indigenous food systems are being revitalized by Indigenous Peoples. IFS recognizes food as sacred, emphasizes self-determination, advocates for decision-making and participation in food systems, and transforms colonial laws, policies, and economies that threaten Indigenous food security (Alabi & Robin, 2022). Many Indigenous communities are leading IFS work by maintaining and revitalizing their urban food systems through a variety of land-based practices and initiatives that promote strong relationships with land and waters, ecological restoration, intergenerational knowledge sharing, community cohesion, healing, and cultural and food knowledges related to traditional and modern food practices (e.g. hunting, fishing, gathering, and growing) (Alabi & Robin, 2022; Skinner et al., 2016; Watson et al., 2022).

### Community context and program description

Initiatives aimed at re-sowing Indigenous food systems are expanding in the Waterloo-Wellington (WW) Region alongside a growing urban Indigenous population. The WW Region is located on the territories of the Anishinaabeg, Haudenosaunee, and Chonnonton Peoples. In 2021, the number of Indigenous Peoples (First Nations, Métis, Inuit) living within the region reached 10,000—a population size that nearly doubled across the preceding 15 years (Region of Waterloo, 2016). To meet the needs of the Indigenous community in this region, two Indigenous organizations, White Owl Native Ancestry Association (WONAA) and the Wisahkotewinowak Collective (WC), are championing Indigenous food sovereignty initiatives in support of food security and community health and wellbeing. WONAA is an Indigenous community-based agency that offers programs and services through a land-based approach to promote healing and wellbeing among urban Indigenous families across generations (WONAA, 2024). As WW Region’s oldest Indigenous organization, established in 1975, WONAA has a long and reputable history within the urban Indigenous community it serves. WC is an urban Indigenous collective that manages several garden sites and the WONAA sugarbush within WW Region, growing both Indigenous (e.g. Haudenosaunee white corn, amaranth) and other produce. Originating as a grassroots initiative in 2016, WC is now a registered non-profit comprised of Indigenous (First Nations and Métis) and settler-allied people whose aim is to build land-based relationships and contribute to the establishment of an urban Indigenous food system in the area (Wisahkotewinowak, 2024).

Given their shared objectives and values, WONAA and WC often work in partnership to support each other in their IFS initiatives. One initiative^1^ they collaboratively operate is the White Owl Food Share (from this point referred to as the Food Share). The Food Share was created in 2020 in response to the COVID-19 pandemic as WONAA and WC observed worsening food insecurity and unemployment in the Region, along with disruptions to their in-person Indigenous programming—an important source of community connection. WONAA and WC also experienced a growth in production, wherein the organizations were growing large volumes of food with no clear distribution process, particularly in the context of COVID-19 isolation measures when community gatherings were restricted. Thus, the food could not be shared through community feasts or less formal interactions.

Recognizing that these urgent challenges could be addressed through a single initiative, WONAA and WC created the Food Share and began distributing locally sourced food baskets to Indigenous households in the Region in August 2020. In the initial design of the initiative, food baskets were distributed by WONAA on a weekly basis, year-round, and contained WC’s available produce or preserves according to the season alongside supplement food items (e.g. eggs, meat, bread) purchased from wholesale food suppliers or donated from local restaurants or bakeries. Baskets were free of charge on a first-come, first-serve sign-up basis and available to be picked-up at WONAA facilities or delivered to participants’ homes. All Indigenous households, regardless of financial need or size, were invited to access the Food Share.

After the first year of the Food Share, WC and WONAA sought to evaluate the initiative’s activities and initial outcomes through a qualitative process evaluation. Within this evaluation, our goals were to assess the Food Share’s implementation, including determining the strengths and weaknesses of the initiative’s design and attending to its context and mechanisms of impact (Moore et al., 2015). We also sought to assess the Food Share’s early outcomes, including identifying initial successes and whether initiative objectives were met (Rossi et al., 2004). In pursuit of these aims, our research objectives were to (1) explore the experiences and perceived initiative meaning of Food Share participants; (2) assess how the Food Share is implemented from the perspectives of operational staff; and (3) gather recommendations for initiative improvement from Food Share participants and operational staff. By evaluating the Food Share and implementing community-specific recommendations generated from this project, WONAA and WC are positioned to support IFS initiatives in the WW Region that strengthen food security, sovereignty, community health, and wellbeing.

## Methods

This project used community-based participatory research (CBPR) methodology, bringing together researchers from the University of Waterloo in partnership with WONAA and WC to design, implement, interpret, and disseminate this evaluation research. CBPR is a collaborative methodology structured by principles of power-sharing, cultural humility (i.e. dedication to self-reflection and responsive learning), and collaboration and equitable partnerships between researchers and communities to strengthen health and wellbeing (Israel et al., 2018). It was carried out by building on existing strengths and resources within communities, co-learning and capacity building among all partners, producing and sharing knowledge for community benefit, incorporating local knowledges and acknowledging the interplay of diverse determinants of health, and committing to long-term engagement in the research process as needed (Israel et al., 2018). This project also adhered to principles of the Ontario Federation of Indigenous Friendship Centres USAI (utility, self-voicing, access, inter-relationality) research framework, which emphasize that research should be “community driven, community relevant, faithful to Indigenous identity, self-voiced, useful, accessible, and relations-based” (OFIFC, 2016, p. 5) to benefit involved urban Indigenous communities. This methodological understanding was incorporated into a program-specific evaluation framework developed through a combination of academic literature, consultation with program leads, and evaluation experience, forming a shared understanding of the initiative and evaluation process.

In alignment with these approaches, the authors of this article represent a team of Indigenous (DS, SP, KP) and non-Indigenous (LP, HN, KS) collaborators who respect each other’s gifts, capacities, and knowledges, which enabled this research to be conducted in a good way. While the lead author (LP) was responsible for carrying out the evaluation, all co-authors participated in the evaluation planning, review of research findings, writing and editing process for publication, with the exception of KP, who joined the project after the evaluation was conducted and supported the writing and editing process. ​

### Recruitment and data collection

With guidance from WONAA and WC, this research applied methodologies and methods that honour Indigenous storytelling and create a relational context for the research team and study participants (Wilson, 2008). As such, individual interviews and a sharing circle were chosen by Indigenous collaborators to collect qualitative data. Recipients of the Food Share program were invited to participate through flyers distributed in food baskets over three weeks in July and August 2021 to reach a diverse clientele who participated in the initiative at varying frequencies and capacities. Interested recipients were then contacted by a member of the research team (LP) by e-mail to arrange an interview. LP conducted eight interviews virtually on Zoom with support from a non-Indigenous undergraduate student at the University of Waterloo. Interviews were audio-recorded and were on average one hour in length. Interviews were supported by a semi-structured interview guide that was collaboratively developed with WC and WONAA and included questions regarding participants’ experiences with the Food Share, what the program means to them, and program recommendations. While demographic data (e.g. age, gender) were not explicitly collected from interviewees, all participants met the recruitment criteria of being adults (i.e. above the age of 18) and self-identifying as Indigenous.

Ten full-time and seasonal operational staff of WONAA and WC were invited to participate in a sharing circle through an e-mail invitation sent by a member of the research team (LP). The sharing circle took place in August 2021 with six participants in attendance. The circle was ceremonially opened and closed by an Indigenous team member (DS) and moderated by a non-Indigenous team member (LP). The sharing circle guide, also developed with WONAA and WC partners, included questions regarding the perceived importance of the Food Share, operational challenges experienced in its pilot year, and recommendations to strengthen its future implementation. Each interview and sharing circle participant received a $50 grocery gift card for their participation. This research received ethics clearance from the University of Waterloo Office of Research (ORE # 41936).

### Data analysis

Audio-recordings from the interviews and sharing circle were transcribed and imported into NVivo 13 qualitative analysis software (Lumivero, 2020). Thematic coding was conducted through an iterative inductive-deductive template analysis process (Fereday & Muir-Cochrane, 2006) adapted from O’Connor and Joffe’s (2020) suggested procedure for intercoder reliability assessment without statistical reliability measurement. Inductive-deductive template analysis takes a semi-structured coding approach by determining *some* themes before undertaking an in-depth full analysis, leaving space for themes grounded in the data to be created (Fereday & Muir-Cochrane, 2006).

This process began with two coders (LP and a non-Indigenous research manager at UW) inductively analyzing a randomly selected batch of 5 transcripts to generate exploratory codes that recognized data-driven meaning before a process of interpretation (Fereday & Muir-Cochrane, 2006). The coders then met to compare their coding decisions and establish consistency and dependability in their interpretation of the qualitative data (Braun & Clarke, 2013), rather than a single true “reliable” meaning (O’Connor & Joffe, 2020). The coders also referenced the evaluation framework to revise their coding decisions and further develop themes. This process established a preliminary coding frame that reflected key theoretical process and outcome evaluation aspects (e.g. initial successes) and data-driven themes. LP then deductively applied this coding frame to all transcripts while further building and refining themes identified. Afterwards, an analysis check-in was scheduled with all members of the research team (Indigenous and non-Indigenous) to validate the interpretation of findings and further refine themes identified, ensuring the data were interpreted through a common conceptual framework (O’Connor & Joffe, 2020) that reconciled data-driven and theory-driven codes. This feedback was incorporated in a final round of analysis by LP, leading to the results presented in this manuscript.

## Results

This manuscript reports three overarching themes that were identified from the interviews and sharing circle data related to program experiences and reflections, process and implementation, and recommendations. While these themes represent over 30 sub-themes related to the process and outcome components of the evaluation, we report the most salient findings here as collaboratively determined by the full research team.

### Initiative experiences and reflections: the Food Share nourishes people through relationships

A shared experience identified by all participants was the Food Share’s prioritization of relationships, which was a key aspect of the initiative’s overall design. Specifically, many participants appreciated the personal contact they had with operational staff, whether through social media or text messaging when making food orders and delivery plans, or in-person conversation during house deliveries. Many participants felt that this relational cornerstone was what made the Food Share accessible and approachable. The Food Share’s relationality also motivated them to continue participating in the initiative because of the sense of connection they felt through consistent, positive interactions with the same staff. The Food Share was easy to use and engage in, and it respected participants’ dignity as they accessed community food support. As one Food Share recipient described:

That’s a strength of this program, the people aspect of it, and it’s not dehumanizing like other food box programs. There’s a relationship there, a connection that doesn’t make you feel degraded for needing help to put food on the table – you’re not just a number picking up a box. (Food Share Recipient 6)

Participants also valued the Food Share because it incorporated important cultural values of reciprocity, love, and responsibility, along with strengths-based approaches to meeting the needs of community members. As a critical community support, this initiative alleviated financial stress for some and strengthened cultural and community connections along the way. As two recipients noted:

I think it’s because we’re all living in this world together and it’s good to keep each other in mind. Some people need help and sometimes it’s just nice to know that you’ve got people out there looking out for you, and that you can look out for a lot of people because we’re all stronger together… [I]t’s kind of what connects us back to our roots. (Food Share Recipient 4)

This is the type of food that is so expensive in the stores, and for you guys to just give it freshly picked, you could just feel the love and, like, the love and growth of that food. The caring of it, and when that stuff comes into my home, I feel good energy. (Food Share Recipient 3)

Relatedly, several recipients discussed their own practices of food sharing that were enabled by the large portions received in the baskets. In these instances, gifting promoted further gifting, as one recipient shared:

So, even though I just live by myself, I’ll make up a big meal and either bring some to work for my co-workers or my family. Yeah, I’m always liking to share because you guys give so much. [Laughs]. You guys are so generous with everything. (Food Share Recipient 3)

Along with human-to-human connections, almost all participants described a significant sense of ecological relationality within the context of the Food Share. For instance, participants expressed their appreciation for receiving local, fresh foods that were cultivated using Indigenous ecological practices and rooted in meaningful relationships with the land. Many articulated their deep appreciation for the Food Share as it connected them, “to place and the people growing it” (Food Share Recipient 8). Another participant noted, “That’s a big part: the relationship with the land and understanding the process and the energy that goes into creating food…recognizing [that’s] how Mother Earth sustains us” (Food Share Recipient 5).

For some, receiving traditional foods through Food Share baskets was especially impactful to wholistic wellbeing, particularly in the context of isolation measures within the pandemic. Traditional foods provided comfort as well as physical, cultural, and spiritual nourishment, as one participant described:

The past year, I’ve had a lot of trouble staying balanced because of COVID – the isolation it entails, but also dealing with the death of my mother. [But] traditional foods help me to feel whole, like it’s nourishing my spirit. (Food Share Recipient 2)

In these instances, traditional foods themselves were relational, as participants described them as contributors to healing and balanced wellbeing.

### Process and implementation: capacity limitations constrain the relational benefits of the Food Share

Although the Food Share fostered great success, the initiative’s implementation was not without its challenges. Basket recipients and operational staff identified several limitations to the initiative’s usability and operation. For instance, personal barriers such as physical limitations prevented some older adult participants from being able to prepare or cut through some of the larger and denser produce provided such as sweet potatoes or cabbage. Many participants also identified their lack of food knowledge or skills that prevented them from using all the produce. This challenge was often amplified by operational inefficiencies, including poor communication. As one participant shared:

One of the challenges is that I don’t always know what’s in the boxes. I have noticed that what is promoted online on Tuesdays doesn’t always reflect what I receive. So, I get stuff I don’t need, and don’t necessarily like, which creates waste…[unless] I figure out who to give [that stuff] to so it doesn’t go to waste. (Food Share Recipient 6)

Food waste was a concern expressed by most participants, which many attributed to the seasonal delivery model. Several times within the Food Share, basket recipients reported receiving too much of a particular food item due to a bumper crop (e.g. okra). Some participants felt distressed because they were unable to consume all of the item, despite trying to think of new and varied ways to make use of and consume it. A few basket recipients addressed this issue by communicating with operational leads and adapting their orders. As Food Share Recipient 3 noted, “I don’t like to waste food, so there will be times where I’ll say ‘I got enough [for the next two weeks].’”.

From an operations standpoint, Food Share staff described capacity limitations among its small team size. This hindered their ability to manage or streamline the work associated with basket distribution. At the same time, some staff were also concerned that expanding their team could impact the relational core of the Food Share. As one facilitator noted:

It’s hard because I feel like the way we have it running is great because it is so small, [which] keeps that ‘food share’ mentality – that more personal aspect of relationship building that makes us unique and who we are. At the same time, I do feel like sometimes we are stretched a little thin. (Operational Staff 1)

The lack of a centralized location and formal infrastructure was also seen by all staff as a significant challenge to the initiative’s coordination. Notably, working across WW Region in geographically distanced garden sites presented a challenge to staff members’ sense of connections to each other and to place. As one staff member described:

There’s also limitations in how we have stuff spread out because we don’t have a building or a [single] location that we work out of as land-based workers. We just don’t have that place which becomes really frustrating [because] of this run around kind of thing. (Operational Staff 2)

Operational staff recognized the benefit of having a central land base to strengthen operations and reinforce relational networks that guide the Food Share.

### Recommendations: enhanced resources, outreach, and infrastructure can strengthen the Food Share

In terms of recommendations, participants identified several ways to address the Food Share’s usability and operations. To enhance usability, Food Share participants recommended the inclusion of recipes in baskets, as well as pre-prepared meals so recipients can make better use of what the Food Share grows and distributes. As one recipient suggested, “There are probably other seniors like me who have more of a challenge doing food preparation, and it’s assistance like that which I’m finding I need more of these days” (Food Share Recipient 1).

Importantly, almost all participants expressed a desire to expand Food Share outreach and include more households that are interested in the initiative. Many recipients also called for additional food-related community programming and activities that could support the Food Share, build on its wholistic health benefits, and fill knowledge and skill gaps. For example, one recipient recommended, “Community wild harvesting events…[or] maybe a canning party where the community can gather and be part of processing the foods” (Food Share Recipient 6). Participants recognized these events as an opportunity to develop community capacity and involvement in local food practices and cultivation, along with reciprocal relationships in community that could support the Food Share or other WONAA and WC programming in the future.

Alongside community capacity and relationships, facilitators also emphasized the importance of securing long-term funding to continue the program, as well as physical infrastructure to structurally centralize the Food Share. As one staff member reflected:

It would be nice [for us workers to] have a place where we could come and hang out and have a coffee [break], creating that more communal space where we connect a little bit differently. And then having that place where we share our food from, where people know there’s more to it than just picking up food. (Program Facilitator 2)

Establishing a central location, secured with sustainable funding, would define a place for the Food Share, where community members can gather and build relationships that form the heart of the initiative.

## Discussion

Grounded in a community-based, collaborative, and relational research approach, this research highlights the important work of an urban Indigenous food security initiative within southwestern Ontario by identifying participants’ Food Share experiences, their reflections on process and implementation of the initiative, as well as their future recommendations. From this evaluation, participants identified the importance of relationships to the initiative, operational capacity limitations, and the need for enhanced resources, outreach, and physical and centralized infrastructure to strengthen the Food Share.

Among the outcomes of this project, a key finding was identifying community relationships as a critical mechanism of impact of the Food Share. Cultural values of relationality, responsibility, respect, and reciprocity that were highlighted within the Food Share are common among Indigenous-led community food programs and initiatives across Turtle Island (Miltenburg et al., 2022). Though Indigenous cosmologies are diverse, these values and ways of being are shared across many Indigenous cultures and they help structure and promote individual and community health and wellbeing (Meissner, 2022). Relationships structure all life, and being in balanced ecological kinships with the land, waters, and more-than-human beings that offer their gifts of food is central to caring for oneself, community, and Mother Earth who sustains us (Malli et al., 2023). Recognizing food as sacred, and accepting these gifts from the earth, fosters a relationship and invites an ethic of reciprocity: a responsibility to accept the gift and to give back (to the land and to others) (Meissner, 2022). Gifting is relationship, reciprocity, respect, and responsibility in action, and through the Food Share, all participants are invited into a communal, ethical space of gifting, whereby relationships are strengthened among community members, growers, distributors, and living beings who share space within the local food system.

Rooted in these cultural values, Indigenous-led care for Indigenous communities is a critical public health approach (Horlick & Chatwood, 2023; Watson et al., 2022). For instance, relationality invites meaningful community involvement, whereby members are engaged in program design and activities, and therefore experience greater ownership, autonomy, and reciprocity as they receive and contribute to community food supports (Timler et al., 2019). These valuable contributions also negate feelings of shame and stigma that many people experience when using mainstream charity food supports such as food banks (Middleton et al., 2018). Further, centring community initiatives in relationality resists colonial denial of Indigenous connections to the land and one another, and this relational approach can be particularly meaningful in urban places that are persistently mischaracterized as settler spaces vacant of Indigenous presence (Congress of Aboriginal Peoples, 2019).

Indigenous-led food supports also often include and prioritize traditional or country foods within their initiatives (Ramirez Prieto et al., 2023). This practice is significant because traditional foods are known to benefit wholistic wellbeing (i.e. they nourish physical, mental, emotional, and spiritual wellbeing), and they (re)connect community members to Indigenous food and knowledge systems that are threatened by colonialism (Malli et al., 2023). By producing, nurturing, harvesting, and sharing a balance of traditional and conventional foods within community, the Food Share helps alleviate some of the financial burden that increases food insecurity risk, while fostering social connections and cultural practices that strengthen Indigenous lifeways and gifting economies (Richmond & Dokis, 2023).

Similar to WONAA and WC, many Indigenous communities and organizations across Canada adapted, expanded, and created new food sharing practices (e.g. Indigenous food banks, community gardens, gifting networks, and school-based programs) to promote food security during the pandemic through Indigenous-led organizing (Ramirez Prieto et al., 2023). In response to the pandemic, some Indigenous communities and organizations applied for and received specialized, temporary funding to support community food initiatives with good effect (Horlick & Chatwood, 2023). However, the need for adequate, long-term funding within Indigenous-led community food initiatives remains a major challenge (Richmond & Dokis, 2023; Watson et al., 2022).

Across the last three decades, government has downloaded the responsibility of many social services in cities (e.g. housing and food supports) to Indigenous organizations, without transferring significant decision-making power and providing adequate and stable funding to support Indigenous groups who lead this work. As such, Indigenous organizations carry the immense task of providing culturally appropriate services to community members without adequate resources, autonomy, and power (Congress of Aboriginal Peoples, 2019). This problem intensifies with a growing urban Indigenous population and increased competition among urban Indigenous organizations positioned to compete against one another over inadequate funds (Congress of Aboriginal Peoples, 2019). Recent research has emphasized these structural and systemic constraints in other service contexts, noting that the COVID-19 crisis highlighted the vital role Indigenous organizational leadership played in identifying and meeting community needs (Howard-Bobiwash et al., 2021).

Recognizing food insecurity as a major threat to Indigenous health and wellbeing and given its root cause of settler colonialism in Canada, many Indigenous communities and research groups across the country have called for approaches that are centred in Indigenous self-determination and considerate of diverse determinants of health. For instance, to promote food security, Indigenous groups have advocated for better protection of the environment and Indigenous food systems therein; wholistic approaches that address socioeconomic factors including poverty, unemployment, and education; ongoing research and education to build public awareness surrounding Indigenous food systems and practices; and strengthened relationships among Indigenous communities and between humans and ecological kin within local food systems (Assembly of First Nations, 2021; Shafiee et al., 2023). In support of Indigenous-led action, government must commit to appropriately funding urban Indigenous organizations in the public health services they provide, and meaningfully involving urban Indigenous Peoples in shaping policies that affect Indigenous food security, food systems, and health and wellbeing (Richmond & Dokis, 2023; Watson et al., 2022).

Since this study was conducted, the Food Share has continued to grow and adapt to community needs in a post-pandemic world. In 2022, the initiative provided food baskets to 60 families on a weekly basis. This increased demand paired with limited funding and a desire to prioritize the relational spirit of the Food Share prompted a shift in the food delivery model in 2023 to in-person community meals and take-home provisions. With the success of this shift, WONAA and WC are now exploring the addition of a Food Pantry to expand the reach of their relational care, furthering the evolution of their initial desire to nourish their community.

## Conclusion

As partnering organizations, WONAA and WC continue to provide Indigenous community members with food security supports through the relational care approach encompassed by the Food Share. Through this relational approach, Indigenous people experience a more wholistic and meaningful sense of being nourished by, with, and through food. In this way, relationships act as a key mechanism of impact within the Food Share. This paper adds to the limited but growing body of literature surrounding Indigenous food security and food-related supports being created and led by urban Indigenous organizations within Canada. Further research specific to urban Indigenous food environments is needed to support Indigenous Peoples who lead work in this area in support of local Indigenous food systems and diverse communities that exist within cities.

## Contributions to knowledge

What does this study add to existing knowledge?

- There is a scarcity of research examining the impact of Indigenous-determined food support programs within urban Canadian centres.
- This study highlights a community-driven, place-based food support initiative by and for Indigenous peoples in an urban Ontario community.
- Study results demonstrate the importance of Indigenous-led relational care approaches in response to increasing food security challenges among urban Indigenous peoples within Canada.

What are the key implications for public health interventions, practice, or policy?

- Indigenous self-determined and relational responses to community food insecurity can support the wholistic wellbeing of Indigenous individuals and communities.
- Sustainable and adequate long-term funding sources for Indigenous-led community food initiatives are critically needed to meet the food security needs of urban Indigenous Peoples within Canada.

## Data Availability

N/A.

## Footnotes

1 We intentionally use the word “initiative” to better describe the activities, effort, and spirit of the Food Share and its context. While the Food Share could be considered a program in evaluation terms, we believe this framing formalizes and thereby constrains the vibrant, relational reality in which this work takes place.

## Code availability

N/A.
